# Positive and negative viral associations in patients with acute respiratory tract infections in primary care: the ECOVIR study

**DOI:** 10.3389/fpubh.2023.1269805

**Published:** 2023-11-24

**Authors:** Hortense Petat, Matthieu Schuers, Christophe Marguet, Xavier Humbert, François Le Bas, Andry Rabiaza, Sandrine Corbet, Bryce Leterrier, Astrid Vabret, Meriadeg Ar Gouilh

**Affiliations:** ^1^Department of Paediatrics and Adolescent Medicine Rouen, Univ Rouen Normandie, Dynamicure INSERM UMR 1311, CHU Rouen, Rouen, France; ^2^Department of General Practice, Univ Rouen Normandie, INSERM U1142, CHU Rouen, Rouen, France; ^3^Department of General Practice, Univ Caen Normandie santé, Caen, France; ^4^Department of Virology, Univ Caen Normandie, INSERM Dynamicure UMR 1311, CHU Caen, Caen, France

**Keywords:** respiratory virus, acute respiratory infections, primary care, respiratory syncytal virus, rhinovirus, respiratory coinfection

## Abstract

**Introduction:**

Acute respiratory infections (ARIs) are the most common viral infections encountered in primary care settings. The identification of causal viruses is still not available in routine practice. Although new strategies of prevention are being identified, knowledge of the relationships between respiratory viruses remains limited.

**Materials and methods:**

ECOVIR was a multicentric prospective study in primary care, which took place during two pre-pandemic seasons (2018–2019 and 2019–2020). Patients presenting to their General practitioner (GP) with ARIs were included, without selecting for age or clinical conditions. Viruses were detected on nasal swab samples using a multiplex Polymerase Chain Reaction test focused on 17 viruses [Respiratory Syncytial Virus-A (RSV-A), RSV-B, Rhinovirus/Enterovirus (HRV), human Metapneumovirus (hMPV), Adenovirus (ADV), Coronaviruses (CoV) HKU1, NL63, 229E, OC43, Influenza virus (H1 and H3 subtypes), Influenza virus B, Para-Influenza viruses (PIVs) 1–4, and Bocavirus (BoV)].

**Results:**

Among the 668 analyzed samples, 66% were positive for at least one virus, of which 7.9% were viral codetections. The viral detection was negatively associated with the age of patients. BoV, ADV, and HRV occurred more significantly in younger patients than the other viruses (*p* < 0.05). Codetections were significantly associated with RSV, HRV, BoV, hMPV, and ADV and not associated with influenza viruses, CoV, and PIVs. HRV and influenza viruses were negatively associated with all the viruses. Conversely, a positive association was found between ADV and BoV and between PIVs and BoV.

**Conclusion:**

Our study provides additional information on the relationships between respiratory viruses, which remains limited in primary care.

## 1 Introduction

Acute Respiratory Infections (ARIs) are mostly associated with viruses and are the most frequent infections, with 0 to 6 ARI per person per epidemic season in temperate regions (1). Although they can occur at any age from childhood onwards, older adult communities and patients with chronic respiratory diseases represent the most vulnerable population, with a high level of healthcare use and mortality in the older adult. This study was conducted to provide a new immunization treatment for Respiratory Syncytial Virus (RSV) (1, 2) and to extend the indication of the flu vaccine to the entire French population, which raises hopes of a major impact on public health. Human Rhinovirus (HRV) is the second major cause of ARI and is known to trigger acute bronchiolitis in infants and severe exacerbations in patients with chronic respiratory diseases (3–6). However, epidemiology and assessment of the burden of these viral infections were provided by emergency units, inpatient stays, or outpatient clinics. This reflects a selected population and the most severe diseases. However, knowledge of the viruses responsible for acute respiratory infections in primary care is limited (7, 8). Studies available in the community focus on a single virus in adults or children (9, 10) or target inappropriate prescribing of antibiotics (11). The development of standardized molecular diagnosis and multiplex Polymerase Chain Reaction (PCR) tests has considerably increased the number of viruses identified and allowed us to compare studies. In addition, these diagnostic tests have improved the frequency of respiratory virus co-detection, which ranges from 25 to 55% (3, 5, 6, 11–13). Although their pathophysiological significance remains unclear, recent data point to a possible competition between viruses, i.e., rhinoviruses and RSV. With the development of immunization research comes the need to improve this knowledge in the community population. Therefore, we conducted a prospective study in primary care settings in patients presenting with symptoms of acute viral respiratory infection (ARI). The viruses responsible for ARI were identified by collecting airway secretions with nasal swabs.

## 2 Materials and methods

### 2.1 Protocol

The ECOVIR project was a prospective, multicentric, non-interventional study developed in Normandy, France, conducted during two viral epidemic seasons (January–April 2019, 12 weeks; October–March 2020, 21 weeks). The design was previously detailed (14). A total of 36 General Practitioner Investigators (GPIs), in eight different healthcare centers, enrolled patients during a medical visit. Inclusion criteria were patients of any age, consulting their general practitioner with symptoms of acute respiratory infection. Exclusion criteria were epistaxis and coagulopathy. Each patient was informed and then examined by a GPI. The GPI categorized the patient as upper or lower acute respiratory infection and performed a nasal swab for the collection of airway secretions in a specific tube. The samples were kept at +4°C in the office and collected twice a week, within the 48 h after the nasal swab application. Commonly, upper ARIs included rhinitis, sinusitis, angina, pharyngitis, laryngitis, and otitis. Lower ARIs included asthma, bronchitis, and community-acquired pneumonia. Flu syndrome was defined by fever, asthenia, cough, and muscular pains. The patients were divided into age groups: infants (0–1 Y), young children (2–5Y), children (6–17 Y), young adults (18–29 Y), medium adults (30–44Y), adults (45–64Y), young senior (65–74Y), and senior (>75Y).

The sampling delay (SD) was defined as the number of days between the first symptoms and the day of swabbing.

### 2.2 Ethics statement

We obtained the approval of the East II protection committee (study reference 15/10/10/63004). Each adult patient and the parent of each child patient was informed and provided their consent. Any additional specific information was given and consent was obtained in patients older than 11 years.

### 2.3 Methods

Samples were kept at four degrees, aliquoted, and processed at the virology laboratory. Nucleic acid extraction was performed on “QIAsymphony” (Qiagen^®^, Hilden, Germany) following the manufacturer’s instructions and then analyzed by NxTag RPP Luminex kit for virological identification. A total of 17 viruses [Respiratory Syncytial Virus-A (RSV-A), Respiratory Syncytial Virus-B (RSV-B), Rhinovirus/Enterovirus (HRV), human Metapneumovirus (hMPV), Adenovirus (ADV), Coronaviruses (CoV) HKU1, NL63, 229E, OC43, Influenza virus (H1 and H3 subtypes), Influenza virus B, Para-Influenza viruses (PIVs) 1–4, Bocavirus (BoV)], and 3 intra-cellular bacteria (*Chlamydophila pneumoniae*, *Legionella pneumophila*, *Mycoplasma pneumoniae*) were targeted. Samples were also tested retrospectively for the presence of SARS-CoV2 nucleic acid using the C-gene Eurobio kit on stored RNA.

### 2.4 Statistical analysis

Quantitative tests were performed using Student-t or Mann–Whitney tests according to the normal distribution. Qualitative tests were performed using Chi2 and Fisher exact tests as needed. Statistical significance was considered at *p* < 0.05. Multivariate analyses were performed using logistic regression; the models the models included continuous variables such as age and SD, and dichotomous variables such as virological results and seasons. The analyses were performed with RStudio^®^ software (version 1.1.456, packages ggplot2, tidyverse). The results were expressed as median [IQ_25–75%_] or number (n) and percentages (%).

## 3 Results

A total of 685 patients were included: 191 and 494 during the first and second seasons, respectively. A total of 17 samples were not able to be tested because of poor quality; 668 patients and samples were analyzed (Table 1).

**Table 1:** Characteristics of the population.

Age groups	All (*n* = 668)	0–1 Y (*n* = 56)	2–5Y (*n* = 67)	6–17Y (*n* = 75)	18–29Y (*n* = 106)	30–44Y (*n* = 122)	45–64Y (*n* = 146)	65–75Y (*n* = 60)	75–94Y (*n* = 36)
Female	409(59)	28(50)	34(50.7)	42(56)	67(63.2)	85(69.7)	89(69.9)	36(60)	24(63.1)
Symptoms n (%)									
Rhinitis	565(84.3)	49(87.5)	63(94.0)	67(89.3)	92(86.8)	101(82.8)	113(79.4)	48(80)	32(84.2)
Fever	279(41.6)	22(39.3)	42(62.7)	38(50.7)	44(41.5)	46(37.7)	61(41.7)	15(25)	11(28.9)
Cough	533(79.6)	45(80.4)	49(73.1)	61(81.3)	83(78.3)	95(77.9)	116(79.4)	60(90)	30(78.9)
Dyspnea	63(9.4)	14(25)	12(17.9)	3(4)	8(7.5)	9(7.4)	22(15.1)	8(13.3)	5(13;2)
Headache (*n* = 548)	208(37.8)	NE	NE	23(30.7)	46(43.3)	52(42.6)	60(41.1)	21(35)	4(10.5)
Diagnosis n (%)									
Upper ARIs	433(64.6)	39 (69.6)	49 (73.1)	45 (60.0)	78 (63.6)	86 (70.5)	83 (56.9)	29 (48.3)	24(63.2)
Lower ARIs	143(21.3)	16(28.6)	17 (25.4)	10 (13.3)	11 (10.4)	14 (13.5)	38 (26.0)	24 (40.0)	13(34.2)
Flu-like syndrome	94(14.1)	1 (1.8)	1 (1.5)	20 (26.7)	17 (16.0)	22 (18.0)	25 (17.1)	7 (11.7)	1 (2.6)
Viruses n (%)									
Negative	227(33.9)	6(10.7)	7(10.4)	18(24)	34(32.1)	53(43.4)	64(43.9)	27(45)	18(47.4)
HRV	203(30.3)	30(53.6)	36(53.7)	23(30.7)	32(30.2)	32(26.2)	35(23.0)	10(16.7)	5(13.1)
Influenza viruses	95(14.2)	2(3.6)	14(20.9)	19(25.3)	16(15.1)	15(12.3)	16(10.9)	9(15)	4(10.5)
CoV	60(9.0)	5(8.9)	3(4.5)	7(9.3)	12(11.3)	13(10.7)	13(8.9)	2(3.3)	5(13.1)
PIV	42(6.3)	6(10.7)	5(7.4)	6(8.0)	8(7.5)	4(3.2)	8(5.4)	5(8.3)	0
RSV	39(5.8)	9(16.1)	2(3.0)	2(2.7)	6(5.7)	2(1.6)	11(7.5)	2(3.3)	5(13.1)
HMPV	34(5.1)	5(8.9)	4(6.0)	7(9.3)	2(1.9)	7(5.8)	3(2.0)	5(8.3)	1(2.6)
ADV	18(2.7)	6(10.7)	10(14;9)	1(1.3)	0	0	1(0.7)	0	0
BoV	12(1.8)	6(10.7)	4(6.0)	0	0	1(0.8)	1(0.68)	0	0
Codetection	53(7.9)	16(28.6)	15(22.4)	8(10.7)	4(3.8)	4(3.3)	6(4.1)	0	0

Upper ARIs included rhinitis, sinusitis, pharyngitis, angina, laryngitis, and otitis. Lower ARIs included asthma, bronchitis, and community-acquired pneumonia. NE, not evaluable. Multiplex PCR test results: among the 668 samples tested (97.5% patients), 443 (66%) were positive for at least one virus (Table 2). The median [IQ_25-75_] age and SD of patients with negative or positive samples were, respectively, 45 [1–92] years and 27 [0.3–94] years (*p* < 0.0001), and 3 [2-5] days and 2 [2-8] days. The multivariate analysis confirmed that negative samples were independently associated with age by year (*p* < 0.00001) and with the SD by day (*p* < 0.00001) according to the onset of symptoms.

**Figure 1:**
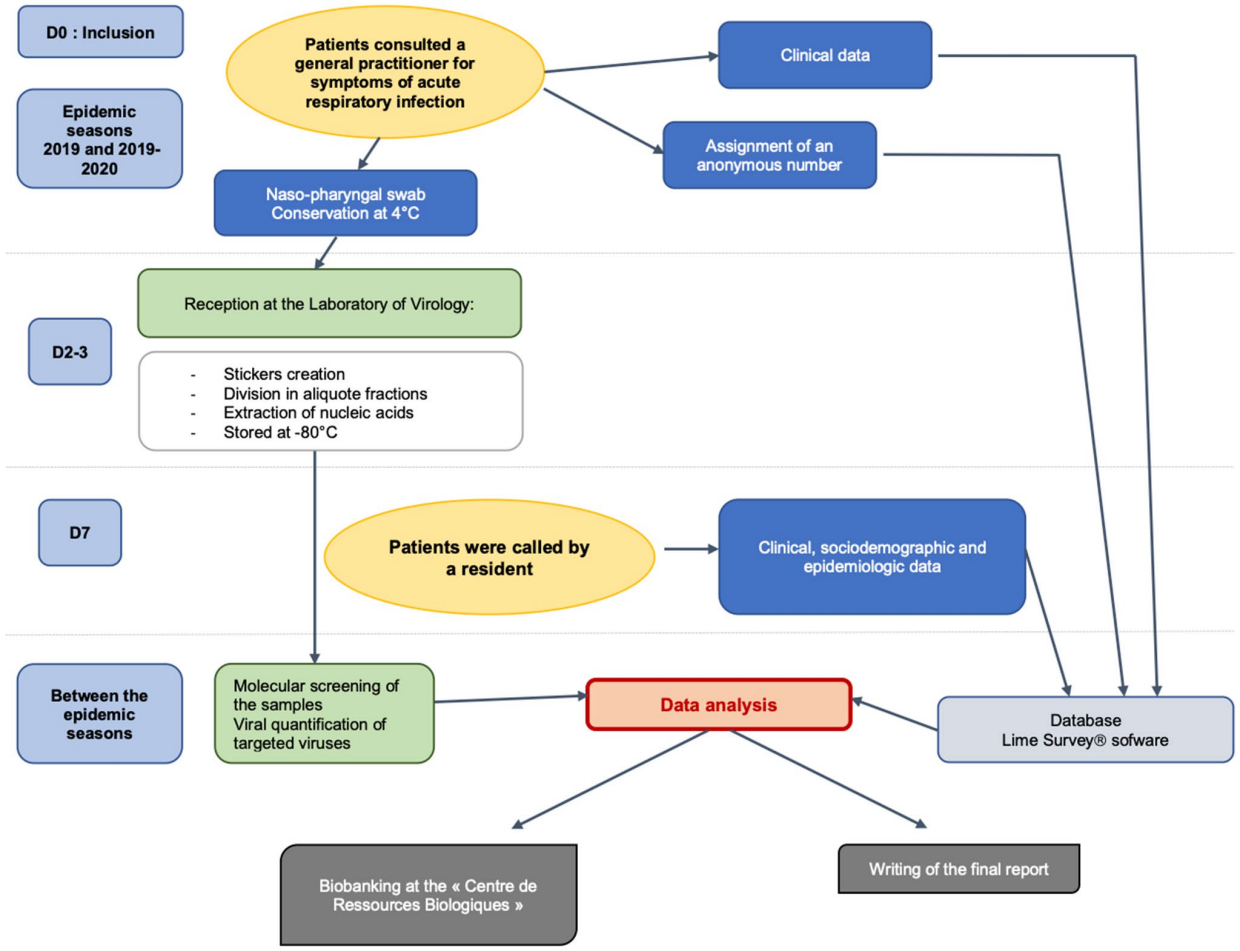
Workflow of ECOVIR study. Adapted from Ordóñez-Mena et al. (16).

The median IQ_25-75_ age of the patients and SD were 32 [12; 54] years and 3 (2–6) days, respectively. Upper ARIs were the most frequent diagnosis, with a comparable distribution in each age group. The frequency of flu-like syndrome was lower at both extremities of life compared to other age groups (Figure 1).

Among the positive samples, the prevalence of each virus was as follows: 46% HRV (*n* = 203), 21% Influenza viruses (*n* = 95), 14% CoV (*n* = 60), 9% PIVs (*n* = 42), 9% RSV (*n* = 39), 8% hMPV (*n* = 34), 4% ADV (*n* = 18), and 3% BoV (*n* = 12). The distribution of the viruses differed according to the age of patients (*p* = 0.0001). The median [IQ_25–75_] ages were as follows: 43 [27–61] years for negative samples; 22 [3–44] years for HRV; 28 [11–53] years for influenza viruses; 34 [17.5–54] years for CoV; 20 [5–51] years for PIVs; 30 [2–60] years for RSV; 24.5 [4–52] years for hMPV; 2 [0.8–3] years for ADV, and 1.9 [1.4–3.5] years for BoV. Only SD of influenza positive samples differed by a shorter time than that observed with the other viruses (*p* < 0.0001). RSV (OR = 3.1, IC_95%_ [1.6;6.0], *p* = 0.0005) and BoV (OR = 3.8, IC_95%_[1.2;12.0], *p* = 0.015) were associated with lower ARIs compared to upper ARIs. Influenza viruses and HRV were positively (*p* < 0.0001) and negatively (*p* < 0.0001) associated with flu-like syndrome, respectively.

**Figure 2:**
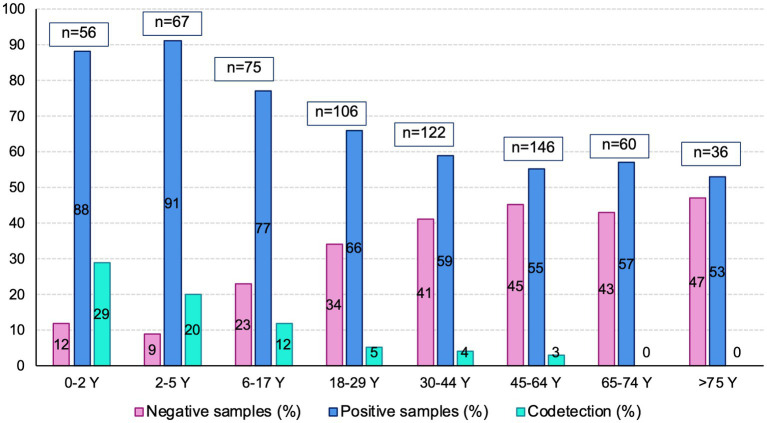
Codetections, negative and positive samples according to age groups.

Viral codetections and associations between the viruses: 53 samples (7.9%) were positive for at least two viruses. The median age of patients with viral codetection was 3 [1.4–19] years compared with 29 [9–54] years for patients infected without codetection (*p* < 0.0001; Figure 2). Viral codetections were found at 2% (*n* = 1) in October, 15% (*n* = 8) in November, 7% (*n* = 4) in December, 32% (*n* = 17) in January, 11% (*n* = 6) in February, 24% (*n* = 13) in March, and 7% (*n* = 4) in April (*p* = 0.003). The risk of viral codetection was significantly related to the viruses (Table 2); it was significantly high for HRV, RSV, hMPV, and ADV and 100% for BoV. Codetections were also independently related to age but not months. The associations between the viruses are displayed in Table 3. HRV and influenza viruses negatively interfered with other viruses, except AdV and BoV. The multivariate analyses highlighted variation in the associations between the viruses according to age, seasons, and SD: HRV was negatively associated with influenza viruses, PIVs, and CoV, influenza viruses were negatively associated with HRV and CoV, and BoV positively interfered with PIVs. Influenza viruses remained the only ones negatively associated with the length of SD.

**Table 2:** Viral codetections according to the viruses.

		Univariate analysis		Multivariate analysis	
Viruses	Codetections n (%)	OR [CI_95%_]	*p*	OR [CI_95%_]	*p*
HRV (*n* = 203)	37 (18)	3,1[1.7; 5.9]	0.002	3.4 [1.9; 5.8]	<0.001
Influenza viruses (*n* = 95)	13 (14)	1,2 [0.6; 2.4]	NS		NS
CoV (*n* = 60)	9 (15)	1.4 [0.6; 2.9]	NS		NS
PIVs (*n* = 42)	9 (22)	2.2 [1; 4.9]	0.04		NS
RSV (*n* = 39)	10 (26)	2.9 [1.3; 6.3]	0.005	4.0 [2.1; 7.7]	<0.001
hMPV (*n* = 34)	13 (38)	5.7 [2.6; 12.2]	< 0.001	6.1 [3.1; 11.8]	<0.001
AdV (*n* = 18)	12 (67)	18.7 [6.7; 51.4]	< 0.001	6.5 [3.0; 14.3]	<0.001
BoV (*n* = 12)	12 (100)	NC	–		NC
Age (years)	–	–	–	0.11 [0.03;0.37]	

NS, not significant; NC, Not Calculable. The model of logistic regression included the viruses, age, SD, and seasons.

**Table 3:** Associations between the viruses and risk of viral coinfection.

	RSV	Influenza viruses	hMPV	PIV	ADV	CoV	BoV	HRV
RSV *n* = 39	–							
Influenza viruses *n* = 95	1 0.09 [0.01–0.64] ***p* = 0.006**	–						
hMPV *n* = 34	2 NS	2 0.21 [0.05–0.90] ***p* = 0.02**	–					
PIVs *n* = 42	0	1 NS	0	–				
ADV *n* = 18	0	2 NS	1 NS	1 NS	–			
CoV *n* = 60	1 NS	2 0.11 [0.03–0.45] ***p* < 0.0001**	2 NS	0 NS	1	–		
BoV *n* = 12	0	1 NS	2 NS	3 NS	3 9.22 [2.26–37.5] *p* = 0.009	1 NS	–	
HRV *n* = 203	7 0.23 [0.1–0.53] *p* = 0.0002	6 0.05 [0.02–0.12] *p* < 0.0001	7 0.28 [0.12–0.66] *p* = 0.0014	6 0.17 [0.07–0.42] *p* < 0.0001	9 NS	4 0.06 [0.02–0.18] *p* < 0.0001	6 NS	–

The results show the number of viruses, Odds ratio (OR and _95%_ CI), and probability (*p*).

## 4 Discussion

This study reported data on the respiratory viruses encountered in patients attending GP practices, which remains seldom studied. The aim was to focus on the viral co-detections in a community cohort. This cohort comprised mainly adults aged from 18 to 64 years, a few older adults (14%), and 28% children. All patients attended for ARIs, mainly upper infections, and none were referred to hospital. Thus, this cohort represents the daily activity of GPs. Negative samples were found in one to three patients. Their frequencies increased independently with age and the SD in relation to the onset of symptoms. In children aged between 2 and 6 years, the rate of negative samples was 11%. This was 5 times higher than we reported earlier in infants with bronchiolitis at emergency departments [Guérande study; (5)]. Few primary care studies included comparable profiles of children with mainly upper ARTIs. They reported higher rates of undetected viruses than we did, both at a primary care level (26–28%) (1, 17) and in outpatients (28 to 42%) (4). The rate of negative samples doubled from the age of 6 years and quadrupled from the age of 40 years, then remained stable around 40–45%. This is comparable with those reported in a household (1) and in LRTI cohort studies (8, 18) but was higher than those reported in a “coughing” population (16). The rates of undetected viruses appeared more dependent on the selection of the children than that was in adults. In three studies from primary care (15, 19, 20) and one study from emergency department (21) children were selected with acute cough and acute lower respiratory symptoms. The frequency of negative samples varied from 36 to 45%. An observed rate of 14% was, conversely, found in children less than 6 years old in a recent study (21), contrasting with a very high rate of 64% in those older than 6 years old. In children with influenza-like illness, the prevalence of negative samples was, conversely, the highest in the youngest age group of 0–4 years old (31%) and strongly decreased in the older adult (22).

One patient out of three was infected with HRV, which is the most encountered virus in other primary care studies. This was independent of the selection of the population, except for infant cohorts (2, 7, 18, 19, 22). The other viruses were, by decreasing frequency, Influenza, CoV, PIV, RSV, hMPV, ADV, and BoV. In this study, the chance to detect HRV, PIVs, and AdV decreased with aging. This was, however, not constantly observed for PIVs and AdV, as was previously reported for HRV (4, 21, 22). Interestingly, RSV was not associated with the age of the patient. This might be dependent on the part of infants in the studied population (4, 22). However, in primary care, this meant that RSV concerned all the patients attending their GP practice, which is underestimated. Influenza was the only virus dependent on the SD, suggesting a short delay in consultation regarding severe symptoms. Influenza viruses, RSV, and AdV were strongly associated with the winter season, and other viruses were less dependent on the season.

Viral codetections represented 8% of samples, which is slightly higher than the reported range of 4–7% (22, 23) but less than the 16 to 55% observed in hospitalized patients (5, 24). In this population, we found a decrease in the level of coinfections with age (22, 24), which corroborated the lack of viral immunity in the older adult (25). Codetections also depended on the associations between viruses, while seasons did not. HRV represented two-thirds of coinfections. Whether a high prevalence of HRV has previously been reported through co-infections (8, 22), CoV (8), ADV (1, 4), and Influenza (22, 26) were also reported as the most frequently associated viruses with codetections. HRV and Influenza were negatively associated with all the viruses, except AdV and BoV. The only positive associations were between AdV and BoV and influenza viruses and BoV. This suggests these viral infections can be facilitated by a viral co-infection. In addition, RSV and influenza viruses were significantly not codetected with coronaviruses. Two previous studies reported comparable negative associations between the respiratory viruses (24, 26) but with slight differences. This might be explained by the variations in epidemic peaks from year to year (27), by the selected population (type of disease, age), the site of care (hospital, outpatients, or primary care), and the country, as viruses differed in their duration of circulation in different countries (28). Despite the high frequencies of co-infections between HRV and RSV in infants (5), the negative interference between these two viruses has been documented by temporality studies during the pandemic (29) or in infants protected by palivizumab (30). A comparable relationship was reported with influenza and HRV or RSV in several studies (31, 32). Therefore, viral co-detections are still difficult to interpret. They might be either infections occurring within a short period of time with active viral replication for both viruses or sequential infections with the persistence of one of the two viruses whose virulence is attenuated corresponding to a possible interference between them. Nevertheless, two recent studies provided contradictory data by involving the interferon pathways. Wu et al. demonstrated that HRV attenuated the virulence of influenza A in accordance with previous clinical observations. Eissaidi et al. found that RSV and influenza reduced HRV replication, while HRV had no effect on RSV and influenza replication, regardless of the timing of co-infection (33).

Because of the pandemic, the study was stopped and the expected number of 1,000 included patients was not achieved. As specified, SARS-CoV2 was detected in only two patients, confirming that we stopped before the pandemic and any effect on the viruses’ circulation. In addition, only viruses were researched and analyzed in this study. Although the Multiplex PCR enabled the detection of three intracellular bacteria, only three were found without any co-infection, and the three patients were excluded. Therefore, we could not withdraw possible interactions of bacteria in the association of viruses. The strength of this study is that it shows the acceptability and feasibility of nasal swabs, with little failure, in a prospective study in primary care.

## 5 Conclusion

At a time when new immunization treatments are available to the community and many others are in development, this prospective study has provided new and complementary data on the circulation of the virus in the community during epidemics. Our results suggest that viral clearance, with the exception of influenza, is accelerated in adults and the older adult, which does not facilitate the study of viral epidemiology in the community. In addition, we observed that the diversity of viruses detected may or may not change according to the age of patients. Finally, the presence of viral coinfections and their negative or positive associations is likely to pose a challenge for future immunization. This suggests a possible replacement of eradicated viruses by others. In conclusion, we need further epidemiology studies in primary settings to prepare for possible emergent viral epidemics.

## Data availability statement

The raw data supporting the conclusions of this article will be made available by the authors, without undue reservation.

## Ethics statement

The studies involving humans were approved by East II protection committee, France. The studies were conducted in accordance with the local legislation and institutional requirements. Written informed consent for participation in this study was provided by the participants’ legal guardians/next of kin.

## Author contributions

HP: Conceptualization, Formal analysis, Investigation, Methodology, Resources, Software, Supervision, Validation, Visualization, Writing – original draft. MS: Investigation, Writing – review & editing. CM: Conceptualization, Methodology, Validation, Writing – review & editing. XH: Investigation, Writing – review & editing. FL: Investigation, Methodology, Writing – review & editing. AR: Investigation, Writing – review & editing. SC: Methodology, Software, Writing – review & editing. BL: Formal analysis, Writing – review & editing. AV: Conceptualization, Methodology, Supervision, Writing – review & editing. MA: Conceptualization, Formal analysis, Methodology, Validation, Writing – review & editing.

## GPIs and healthcare centers

- Maison de santé Saint Eloi, Rouen, France: Dr. Aude Le Floch, Dr. Stéphane Le Friec, Dr. Marianne Laine, Dr. Lucille Pellerin, Dr. Marie Rigal-Lashkar, Dr. Barbara Vatin.
- Maison de santé du Pays Neufchâtelois, Neufchatel En Bray, France: Dr. Florence Calero, Dr. Hélène Comont, Dr. Christophe Guillerm, Dr. Claire Houle, Dr. Vincent Lemarie, Dr. Sophie Ozanne, Dr. Anne-Sophie Pimont, Pr Matthieu Schuers, Dr. Olivier Wermester.
- Maison Médicale Amstrong, Grand Quevilly, France: Dr. Caroline Jouen, Dr. Jean-Yves Maunoury, Dr. Elisabeth Mauviard, Dr. Yveline Sevrin, Dr. Charlotte Vianney.
- Pôle de santé du Port, Granville, France: Pr François Le Bas, Dr. Alexis Plunet
- Pôle de santé de Villedieu, Villedieu-Les-Poeles, France: Dr. Olivier Bataille, Dr. Nicolas Boyer, Dr. Philippe Sabathier, Dr. Thomas Trenier.
- Cabinet médical Colombelles, Colombelles, France: Dr. Elise Barbe-Delrue, Dr. Philippe Grujard, Dr. Jeanne Le Bas, Dr. Stéphane Vallayes.
- Pôle santé de Vaucelles, Caen, France: Dr. Xavier Humbert, Dr. Grégory Daireaux, Dr. Charlotte Milcent.
- Maison médicale Deauville Côte Fleurie, Deauville, France: Dr. Andry Rabiaza, Dr. Nicolas Sainmont, Dr. Laurent Simon.
